# A Single Mutation in the PB1-F2 of H5N1 (HK/97) and 1918 Influenza A Viruses Contributes to Increased Virulence

**DOI:** 10.1371/journal.ppat.0030141

**Published:** 2007-10-05

**Authors:** Gina M Conenello, Dmitriy Zamarin, Lucy A Perrone, Terrence Tumpey, Peter Palese

**Affiliations:** 1 Department of Microbiology, Mount Sinai School of Medicine, New York, New York, United States of America; 2 Influenza Division, National Center for Immunization and Respiratory Diseases, Centers for Disease Control and Prevention, Atlanta, Georgia, United States of America; 3 Department of Medicine, Mount Sinai School of Medicine, New York, New York, United States of America; University of Wisconsin-Madison, United States of America

## Abstract

The proapoptotic PB1-F2 protein of influenza A viruses has been shown to contribute to pathogenesis in the mouse model. Expression of full-length PB1-F2 increases the pathogenesis of the influenza A virus, causing weight loss, slower viral clearance, and increased viral titers in the lungs. After comparing viruses from the Hong Kong 1997 H5N1 outbreak, one amino acid change (N66S) was found in the PB1-F2 sequence at position 66 that correlated with pathogenicity. This same amino acid change (N66S) was also found in the PB1-F2 protein of the 1918 pandemic A/Brevig Mission/18 virus. Two isogenic recombinant chimeric viruses were created with an influenza A/WSN/33 virus background containing the PB1 segment from the HK/156/97: WH and WH N66S. In mice infected with WH N66S virus there was increased pathogenicity as measured by weight loss and decreased survival, and a 100-fold increase in virus replication when compared to mice infected with the WH virus. The 1918 pandemic strain A/Brevig Mission/18 was reconstructed with a pathogenicity-reducing mutation in PB1-F2 (S66N). The resultant 1918 S66N virus was attenuated in mice having a 3-log lower 50% lethal dose and caused less morbidity and mortality in mice than the wild-type virus. Viral lung titers were also decreased in 1918 S66N–infected mice compared with wild-type 1918 virus–infected mice. In addition, both viruses with an S at position 66 (WH N66S and wt 1918) induced elevated levels of cytokines in the lungs of infected mice. Together, these data show that a single amino acid substitution in PB1-F2 can result in increased viral pathogenicity and could be one of the factors contributing to the high lethality seen with the 1918 pandemic virus.

## Author Summary

PB1-F2 is the most recently discovered protein produced by the influenza A virus. It has been previously shown that PB1-F2 is present in the mitochondria, where it induces cell death; our laboratory has demonstrated that PB1-F2 is a contributor to pathogenesis in the mouse model of infection. To study PB1-F2 further, we examined highly pathogenic strains of avian influenza virus and located an amino acid change that seemed to be associated with increased death in mice. We studied this amino acid change in PB1-F2 at position 66 in two different viruses. A recombinant virus that has a *PB1* gene from an H5N1 virus was used as well as a fully reconstructed 1918 pandemic virus. In this study, we show that a mutation in PB1-F2 found in highly pathogenic influenza A virus isolates causes nonpathogenic viruses to induce disease in mice. In addition, we show that the increased pathogenicity is associated with higher levels of virus and cytokines in the lungs. We conclude that PB1-F2 does affect pathogenicity, and that position 66 seems to play an important role in contributing to the effects of PB1-F2 in the mouse model.

## Introduction

Influenza A virus causes 300,000–500,000 deaths worldwide each year, and in pandemic years, this number can increase to 1 million (in 1957–1958 ) or as high as 50 million, as was seen in 1918–1919 [1–3]. More recently, H5N1 highly pathogenic avian influenza viruses have generated great concern regarding their potential to cause a pandemic. H5N1 infections in humans were seen in Hong Kong in a small outbreak in 1997 that resulted in 18 human infections and six fatalities, and since 2003, 309 human cases of H5N1 have been confirmed with a 61% fatality rate (6/1/07) [4–7]. Recent work on these viruses has aimed to elucidate the virulence factors that account for the severe illness observed in humans and mice [4,8–12].

The viral *PB1* segment is of particular interest, since, in addition to the glycoprotein genes, the *PB1* gene was the only other segment that was exchanged in the pandemic viruses of 1957 and 1968 [13]. Introduction of a novel *PB1* gene into the 1998 swine reassortant viruses further implicates the role of this gene in the pathogenesis of (animal) influenza [14]. Moreover, while changes in the surface glycoproteins allow the viruses to overcome the preexisting humoral immune response, they may not be solely responsible for the high virulence of the pandemic influenza viruses. In particular, the 1918 pandemic was associated with significantly higher morbidity and mortality than the subsequent pandemics [15]. Recent reconstruction of the 1918 virus has confirmed that the viral polymerase from the 1918 influenza virus is required for full pathogenicity of the recombinant 1918 virus in mice [16]. In fact, substitution of the viral polymerase genomic segments with those of the modern H1N1 strain severely attenuated the virus in mice [16]. Recent identification and characterization of a novel influenza virus protein called PB1-F2 encoded by the *PB1* gene introduced a potential virulence factor that could play a role in pathogenesis of infection with pandemic influenza viruses and explain the selection of the *PB1* gene in these viruses [17].

The influenza virus PB1-F2 is a 90–amino acid (aa) protein that is associated with the induction of cell death. The protein directly permeabilizes mitochondria, resulting in the dissipation of the mitochondrial membrane potential and the release of cytochrome *c* [17–19]. We have previously shown that PB1-F2 contributes to viral pathogenesis in the mouse model and wanted to further investigate whether the PB1-F2 proteins encoded by highly pathogenic viruses have conserved mutations in their aa sequence that are associated with pathogenicity [20]. We chose to study the PB1-F2 proteins of the Hong Kong 1997 H5N1 viruses that caused an outbreak in humans. Characterization of the isolated viruses in mice revealed that the viruses could be subdivided into three different groups based on the pathogenicity phenotype: high-virulence, intermediate-virulence, and low-virulence [21]. Further studies provided molecular correlates of pathogenicity in the high-virulence group, though such studies were not conducted for the PB1-F2 protein [22].

Herein, we assess the contribution of the PB1-F2 protein to the pathogenicity of a highly pathogenic H5N1 virus and the 1918 pandemic strain virus. An alignment of the aa sequences of isolates from the Hong Kong 1997 H5N1 outbreak revealed that a mutation, N66S, was associated with high pathogenicity phenotype in mice. Using a recombinant A/WSN/33 virus with the *PB1* segment of A/HK/156/97, we observed increased morbidity and mortality of mice infected with a virus that contained the N66S mutation. In addition, infection with the reconstructed A/Brevig Mission/18 virus, which has an S at position 66, resulted in increased pathogenicity when compared with a reconstructed A/Brevig Mission/18 virus in which position 66 was changed to N [16]. We thereby show that PB1-F2 proteins from highly virulent viruses can contribute to pathogenicity, and identify a single aa change that confers a virulent phenotype in mice.

## Materials and Methods

### Cell Lines

Madin Darby canine kidney (MDCK), 293T, and A549 cells were obtained from ATCC (http://www.atcc.org/) and were maintained in MEM and DMEM culture media (Gibco, http://www.invitrogen.com/), respectively, supplemented with 10% fetal calf serum (Hyclone, http://www.hyclone.com/) and penicillin/streptomycin (Gibco).

### Constructs and Cloning

The pPolI vectors encoding viral genomic RNA of the WSN strain have been described previously [23]. The *PB1* gene of the A/HK/156/97 virus was reverse transcribed from purified genomic RNA, amplified by PCR with *PB1* segment-specific primers, and cloned into the pPolI vector. The cloning of genes for A/Brevig Mission/18 has been described previously [16]. To generate pPolI vectors encoding the N66S PB1-F2 mutants, the pPolI vectors encoding the A/HK/483/97 *PB1* or A/Brevig Mission/18 *PB1* were subjected to site-directed mutagenesis using the Stratagene Quick-Change mutagenesis kit (Stratagene, http://www.stratagene.com/). Sequences of each construct were confirmed by automated sequencing performed at the Mount Sinai sequencing core facility.

### Reverse Genetics for Recombinant Viruses

The reverse genetics technique for the generation of recombinant influenza viruses has been described previously [23]. Briefly, 293T cells were transfected with eight pPolI vectors encoding the viral genomic RNA segments and four pCAGGS protein expression vectors encoding the subunits of viral polymerase and the nucleocapsid protein. The transfected 293T cells were cocultured with MDCK cells, and virus released into the supernatant was isolated by plaque purification on MDCK cells. The presence of the introduced mutations was confirmed by reverse transcription and sequencing of the *PB1* genes of the newly generated viruses. Viruses possessing 1918 genes were generated under biosafety level 3 (BSL-3 with enhancements) containment [24] to ensure the safety of laboratory workers, the environment, and the public. All subsequent laboratory and animal work with live virus containing A/Brevig Mission/18 genes also was performed under these high-containment conditions.

### Mouse Experiments

Female C57/BL6 mice 6 to 7 wk old (Jackson Laboratories, http://www.jax.org/) were anesthetized with intraperitoneal injection of 0.07 ml of ketamine/xylazine (0.15 mg ketamine and 0.03 mg xylazine), and infectious virus was diluted in PBS/BSA/PS (phosphate-buffered saline/bovine serum albumin/penicillin and streptomycin) and inoculated intranasally in a volume of 30 μl. To assess virus pathogenicity, groups of four mice were inoculated with appropriate dose and were monitored daily for weight loss over 8 d. Mice that lost more than 25% of their initial body weight were killed according to institutional guidelines and scored as dead. To determine viral replication in the lungs, lungs were collected on days 1, 2, 3, 5, 7, and 8 after infection from 2 (days 1 and 2) or 4 (days 3, 5, 7, and 8) mice from each group and two mice in the PBS group. The lungs were homogenized in PBS using a Dounce homogenizer and processed for virus titering. Virus titers in the supernatant of lung homogenates were determined by plaque assay in MDCK cells.

For 1918 recombinant virus infections, female BALB/c mice, 6 to 7 wk old (Jackson Laboratories) were anesthetized with an intraperitoneal injection of 0.2 ml of 2,2,2-tribromoethanal in tert-amylalcohol (Avertin; Aldrich Chemical Co., http://www.sigmaaldrich.com/), and 50 ul of infectious virus diluted in PBS was inoculated intranasally. The 50% lethal dose (LD_50_) titers were determined by inoculating groups of three mice intranasally with serial 10-fold dilutions of virus. LD_50_ titers were calculated by the method of Reed and Muench [25]. Individual body weights from eight mice were recorded for each group daily and monitored daily for disease signs and death for 14 d after infection. For determination of lung virus titers, 18 additional mice were infected intranasally with the intermediate inoculating dose (10^4^ plaque-forming unit [PFU]) of virus. On days 1–3 and 5–8 after infection, three mice from each group were killed, and whole lungs were removed aseptically and homogenized in 1 ml of sterile PBS. Homogenates were titrated for virus infectivity using a standard plaque assay. The statistical significance of virus titer data was determined by using analysis of variance.

### Cytokine Quantitation

To determine the in vivo levels of cytokine supernatants from the lung, homogenates of the lungs of WH-infected mice were assayed for IFN-γ and TNF-α (assay sensitivity, 2 pg/ml) by use of enzyme-linked immunosorbent assay kits purchased from R&D Systems (http://www.rndsystems.com/).

For high-containment laboratory work with 1918 recombinant viruses, the in vivo levels of cytokine proteins were determined from three individual mice per group. On day 4 after infection, mice were exsanguinated from the axilla and killed, and lung tissues were removed from naive and infected mice. Individual whole-lung samples were immediately frozen at −70 °C. On the day of analysis, tissues were thawed, homogenized in 1 ml of cold PBS, and centrifuged at 150*g* for 5 min. Cytokine protein levels were measured from clarified lung homogenates by the Bioplex Protein Array system [26] (Bio-Rad, http://www.bio-rad.com/) using beads specific for mouse IL-1β, IFN-γ, and TNFα. Cytokine protein levels were measured according to the manufacturer's instructions by fluorescently conjugated monoclonal antibodies in duplicate against a standard curve.

## Results

### Conserved Mutations in Highly Pathogenic Influenza A Viruses

It has been previously shown that H5N1 viruses from the Hong Kong 1997 outbreak fall into three separate pathogenicity phenotypes: low, intermediate, and high [22]. The intermediate phenotype had aa sequence identity with the high-pathogenicity phenotype and all of the previously identified molecular correlates of a high pathogenicity phenotype, but caused a less severe disease in mice. However, PB1-F2 was not examined in the study by Katz et al. because it was not known at the time [22]. Alignment of the 1997 human H5N1 PB1-F2 sequences available in the National Center for Biotechnology Information database (http://www.ncbi.nlm.nih.gov/) revealed several aa changes that separated the high-virulence from the low-virulence groups. These were (low-virulence versus high-virulence) E6D, R53K, N66S, and R75H. These changes were silent in the open reading frame of the *PB1* gene. Alignment of the proteins with other PB1-F2 sequences available in the database revealed that with the exception of the N66S substitution, all of the described mutations were previously present in other influenza viral strains. The N66S mutation was of a particular interest, since it was found only to be present in the highly virulent 1997 H5N1 group, in the PB1-F2 proteins of some avian isolates, and in the 1918 A/Brevig Mission/18 PB1-F2 (Figure 1). Interestingly, the A/HK/156/97 virus (from the intermediate-virulence group), which was previously shown to possess all of the molecular signatures of the high-virulence group, possesses N at position 66 of the PB1-F2 protein.

**Figure 1:**
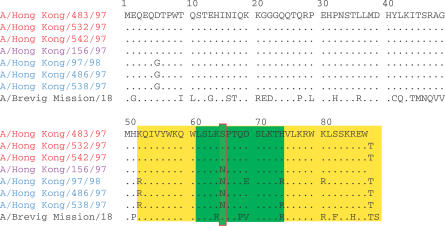
Alignment and Location of N66S Mutation in the PB1-F2 Protein Alignment of human isolates of influenza A viruses from a H5N1 Hong Kong outbreak and from the 1918 H1N1 pandemic. Viruses in red are of the high-pathogenic phenotype, those in purple are of intermediate pathogenicity, and those in blue are of low pathogenicity in mice. Yellow indicates C-terminal region with α-helical structure, and green indicates the minimal mitochondrial targeting sequence.

aa residue 66 resides in the C-terminal α-helical region of PB1-F2. This region is the interacting domain for ANT3 and VDAC1 and contains the mitochondrial targeting sequence, making the C-terminal region essential for the function of PB1-F2 [19,27]. The location of the N66S mutation in the structure of PB1-F2 and its presence in the C-terminal region supports the hypothesis that this aa change could impact PB1-F2′s effects in vivo. We hypothesized that this aa substitution may be responsible for the decreased pathogenicity phenotype observed for the A/HK/156/97 virus. Given these findings, we proceeded to determine whether the PB1-F2 mutation in position 66 (N66S) in the 1997 H5N1 viruses contributed to viral pathogenicity.

### Impact of PB1-F2 aa 66 on Viral Growth and Virulence In Vitro and In Vivo

#### In vitro.

To examine the effect of the mutation in position 66 on the pathogenicity of influenza A viruses, we created recombinant viruses containing either an asparagine (N) or serine (S) at that site of the PB1-F2 protein. A chimeric virus was created in the A/WSN/33 background that contained the A/HK/156/97 *PB1* gene (WH). These viruses were rescued in a BSL-2 environment, making them easier to study. In addition, the WH virus has been characterized in a previous paper [20]. Site-directed mutagenesis was used to introduce the N66S mutation in PB1-F2 without changing the amino acid sequence of the PB1 protein (WH N66S). These viruses were then grown in MDCK cells to determine their growth kinetics in vitro. Cells were inoculated at two different multiplicities of infection (MOIs), 0.1 and 0.001. The two viruses have similar replication kinetics in MDCK cells (Figure 2A). The r1918 and r1918 S66N viruses also have similar growth kinetics in MDCK cells when inoculated at MOIs of .01 and .001 (Figure 2B).

**Figure 2:**
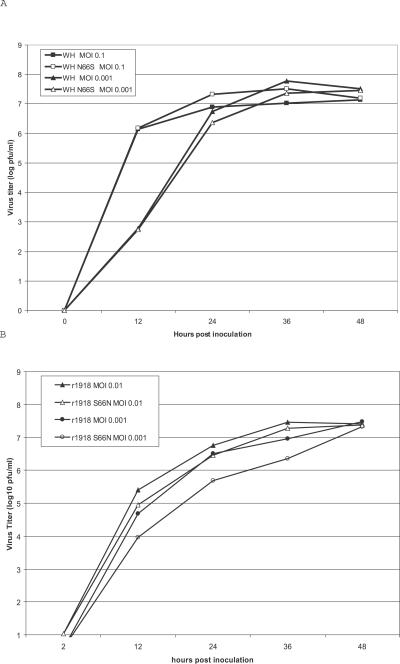
In Vitro Growth Curve of Recombinant Viruses (A) MDCK cells were inoculated at an MOI of 0.1 and 0.001, and virus growth of WH and WH N66S was assessed at the time points indicated. The figure is representative of three similar experiments. (B) MDCK cells were inoculated at an MOI of .01 and .001, and virus growth of r1918 and r1918 S66N was assessed at the time points indicated.

#### In vivo.

Next, 1 × 10^4^ PFU of the viruses were inoculated into mice to determine pathogenicity and viral growth in vivo. Body weights were monitored for up to 8 d after infection. The WH N66S virus caused the mice to start losing weight at day 3, and weight loss continued in all WH N66S–infected mice until day 8, resulting in 50% of the mice succumbing to infection. The WH virus, while causing a slight decrease in weight at day 7, did not cause significant weight loss, and all of the inoculated mice survived the infection (Figure 3A). This difference in pathogenicity is mirrored by the viral replication in the lungs. The WH N66S virus was found to replicate to higher titers in the lungs and exhibited peak virus titers 2 d earlier than the WH virus. WH N66S replication in the lungs was significantly higher than WH on days 2, 5, and 8 after infection, with WH N66S replicating to almost 100 times higher titers on each day (Figure 3B). However, virus levels in the lung were equal on day 7, suggesting that the continued weight loss of WH N66S–infected mice is partially the result of increased cytokine production in the lung. In addition, the high virus titer on day 7 in WH-infected mice corresponds to the mild weight loss seen in Figure 2B. WH N66S–infected mice exhibited slower viral clearance with persisting high viral titers, whereas the WH virus was cleared more effectively from the lung, with 3 out of 4 mice completely clearing the virus by day 8 (Figure 3B). Increased viral load and slowed viral clearance during WH N66S infection could suggest an impaired cellular immune response.

**Figure 3:**
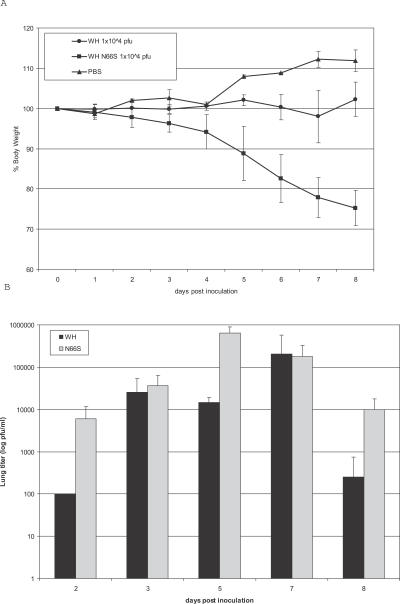
Contribution of PB1-F2 N66S Mutation to Pathogenicity of Recombinant Virus (A) Mice were inoculated with 1 × 10^4^ PFU of virus or PBS, and their weights were recorded every day after infection. (B) Virus titers from lung homogenates were measured from mice infected with WH or WH N66S virus at days 1, 2, 3, 5, 7, and 8 after inoculation. Error bars represent 1 standard deviation.

The 1918 pandemic virus contains an S at position 66 in PB1-F2 corresponding to increased virulence as seen in the A/Hong Kong/483/97 virus. To examine this aa in the context of a fully reconstructed 1918 virus, a single aa change was made (S66N) in PB1-F2 without changing the aa sequence of PB1. To evaluate the virulence and pathogenicity of the 1918 S66N mutant virus, the morbidity (measured by weight loss), virus replication, and LD_50_ titers were determined in BALB/c mice and compared with a group of animals infected with wild-type 1918 virus, previously shown to be highly lethal in mice [28].

As shown in Figure 4A, mice infected with doses of 10^3^, 10^4^, 10^5^, and 10^6^ PFU of the wild-type 1918 virus began to lose weight within 3 d. The mice showed progressive signs of illness, such as ruffled fur and listlessness during the first week of infection before succumbing to infection (LD_50_ = 10^2.5^) by day 10 after inoculation. In contrast to the highly virulent wild-type 1918 virus infection, higher amounts of inoculating virus (10^5^ and 10^6^ PFU) were required to cause severe disease and weight loss among the mice infected with 1918 S66N mutant virus (Figure 4B). Furthermore, the lethality was substantially lower (LD_50_ = 10^5.25^), requiring 500 times more virus than wild-type 1918 virus to kill mice. Infection of mice with the 10^4^ PFU of 1918 S66N mutant virus resulted in lung virus titers, on days 2 and 3 after infection, that were at least 12-fold lower than those of mice infected with the same dose of wild-type 1918 virus (Figure 4C).

**Figure 4:**
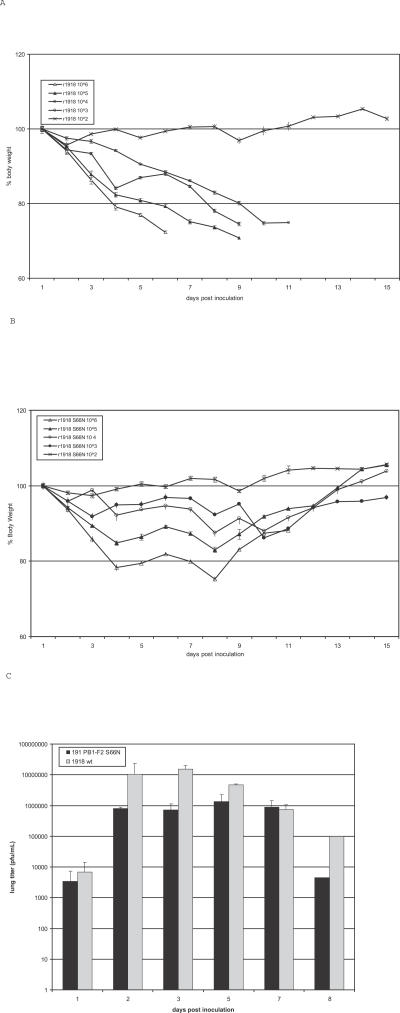
The Effect of the S66N Mutation on Pathogenicity in Mice (A) Eight mice were inoculated with recombinant wt 1918 virus at 10^2^, 10^3^, 10^4^, 10^5^, or 10^6^ PFU, and their weights were measured every day. Average weights are represented in the graph. (B) Eight mice were inoculated with recombinant 1918 S66N virus at 10^2^,10^3^, 10^4^, 10^5^, or 10^6^ PFU, and their weights were measured every other day. Average weights are represented in the graph. (C) Effect of S66N mutation on mouse lung titers in r1918 virus. Virus titers from lung homogenates were measured from 3 mice infected per time point with r1918 or r1918 S66N virus at days 1, 2, 3, 6, 7, and 8 after inoculation. Error bars represent 1 standard deviation.

### Cytokine Dysregulation in the Lungs of Infected Mice

To better understand the increased pathogenicity in the infected mice, we examined the levels of TNF-α and IFN-γ in the lungs. IFN-γ levels were observed to be higher in mice infected with WH N66S virus, especially at days 7 and 8 after infection, when the levels were approximately two times higher than the levels in the WH virus-infected mice (Figure 5A). Levels of TNF-α in the lung also showed significant differences late in infection. At days 7 and 8 after infection, TNF-α levels in mice infected with WH N66S virus had a two times higher increase over levels in WH-infected mice (Figure 5B).

**Figure 5:**
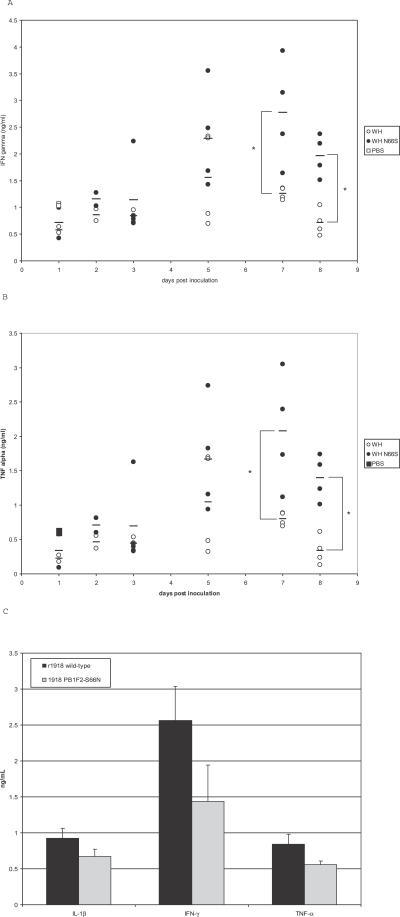
Cytokine Levels in Lungs of Infected Mice (A) IFN-γ enzyme-linked immunosorbent assay was performed on lung homogenates with four mice per time point for each virus and is represented here in ng/ml. *p < .05. (B) TNF-α enzyme-linked immunosorbent assay was performed on lung homogenates with four mice per time point for each virus represented here in ng/ml. *p < .05. (C) Lung homogenates collected on day 4 after inoculation were measured for the levels of IL-1β, IFN-γ, and TNF-α using the Bioplex Protein Array system. All error bars represent 1 standard deviation.

Individual lung tissues were also collected on day 4 after infection from 1918 virus–infected mice. A single timepoint (day 4 after infection) was chosen because it was previously determined that maximal lung cytokine/chemokine levels occurred at this time among mice infected with highly virulent influenza strains [27,28]. Tissues were homogenized and lysates were assayed for cytokines by the Bioplex Protein Array system. Determination of IL-1α, IFN-γ, and TNF-α levels demonstrated that these cytokines were produced above their constitutive levels 4 d after infection with both 1918 S66N mutant and wild-type virus (Figure 5C). All three cytokines were detected at significantly higher levels (*p* ≤ 0.5, analysis of variance) in 1918 wild-type–infected than in 1918 S66N mutant–infected mice. Together, these data indicate that PB1-F2 may play a role in immunomodulation, especially later in infection during viral clearance.

## Discussion

Previous studies by our lab have shown that PB1-F2 contributes to the pathogenesis of the influenza A virus [20]. When expression of PB1-F2 was knocked out of a moderately virulent virus in mice, there was a significant loss in pathogenicity, indicating that PB1-F2 plays an important role in virulence [20]. In the present study, we show that a single aa change in PB1-F2 from highly virulent viruses increases pathogenicity in mice and modulates the immune response. It has been proposed that PB1-F2 causes apoptosis of immune cells, which may lead to decreased antigen presentation and a decrease in the adaptive immune response [17]. Humans infected with highly pathogenic viruses consistently have decreased lymphocytes and impaired immune response to influenza virus infection [4,8,10,21,29,30]. We wondered if these effects could be caused in part by PB1-F2. In this study, we provide evidence that PB1-F2 does contribute to the high pathogenicity phenotype and that the N66S mutation, also found in the 1918 H1N1 virus, contributes to virulence in highly pathogenic viruses.

After aligning the PB1-F2 sequences from H5N1 viruses that exhibited high- and low-pathogenicity phenotypes, a single aa change was found to correlate with high pathogenicity. The location of the N66S mutation also made it an excellent candidate for affecting the proapoptotic function of PB1-F2. Position 66 is in the α-helical structure of PB1-F2, in the mitochondrial targeting sequence. The location of aa 66 in the C-terminal mitochondrial targeting sequence of the protein could affect PB1-F2 interactions with ANT3 and VDAC1, potentially increasing the induction of apoptosis by PB1-F2 [19].

Recombinant A/WSN/33 viruses were created to specifically examine the effects of the N66S mutation during viral infection. The recombinant virus WH has decreased pathogenesis in mice compared with that of A/WSN/33 (unpublished data), likely due to the mismatched polymerase genes, resulting in less efficient replication in the host. The N66S mutation within the PB1-F2 protein partially reversed this attenuating effect.

Within a natural setting, the presence of a “virulent” PB1-F2 may be important when influenza viruses cross species barriers or when new pandemic strains are generated by reassortment. In fact, the *PB1* gene has been one of the segments found to reassort to create the pandemic strains of 1957 and 1968, potentially giving these viruses a more pathogenic PB1-F2 and thus a higher virulence [13]. It is possible that the PB1-F2 protein could allow a newly reassorted virus to replicate in a new host efficiently enough to spread, and develop mutations to create a more efficient polymerase complex. In addition, influenza surveillance data shows that in recent history (1970 onward), H3N2 infections cause almost 14 times the number of influenza related deaths than H1N1 infections and are associated with a higher epidemic severity index (as measured by the rate of increase in pneumonia and influenza mortality) [31–33]. Interestingly, recent H1N1 isolates contain a truncated PB1-F2, which possibly plays a role in their decreased virulence [19,34]. The mutation we investigate here is not currently found in recent H5N1 isolates; however, it is possible for those viruses to acquire the mutation either through the error-prone RNA polymerase or through reassortment with a virus that contains the N66S mutation.

The observation that the WH N66S virus grew to higher titers in the lung and persisted at high titers for a longer time than the WH virus supports the role of PB1-F2 in allowing for increased replication. This may also explain the impairment of viral clearance in the mice infected with WH N66S. In addition, the 1918 wt virus showed higher lung titers and slower viral clearance when compared with the 1918 S66N virus. We suspect that the delay in viral clearance due to expression of PB1-F2 protein may allow for prolonged viral replication and development of irreversible pulmonary immunopathology, the findings observed with highly pathogenic influenza strains. CD8^+^ T cells are mainly responsible for viral clearance in the host, and it is possible that their function could be impaired by PB1-F2 [35,36]. In support of this, we observed that the WH N66S and wt 1918 viruses caused a significant increase in IFN-γ and TNF-α cytokine production over the WH and 1918 S66N viruses, respectively. Whether this change in cytokine levels is through the direct action of PB1-F2 or through its impact on viral replication in the lung is difficult to determine. However, the cytokine dysregulation is of special interest because it has been associated with both H5N1 and 1918 H1N1 virus infections. In previous studies, cytokine dysregulation was associated with high virulence and death in animal models [29,37]. Our study supports these findings and suggests that PB1-F2 could be one of the factors contributing to the cytokine dysregulation seen in H5N1 virus–infected patients and 1918 H1N1 virus–infected animals [4,37].

## Abbreviations

aa: amino acid
LD_50_: 50% lethal dose
MDCK: Madin Darby canine kidney
MOI: multiplicity of infection
PBS: phosphate-buffered saline
PFU: plaque-forming unit
